# Optic Neuritis As the Initial Presentation of Syphilis

**DOI:** 10.7759/cureus.83704

**Published:** 2025-05-08

**Authors:** Bruno Bonito, Joana Cartucho, Monica F Silva, Inês Ferreira Maia, Maria do Rosário Ginga

**Affiliations:** 1 Internal Medicine, Centro Hospitalar Barreiro Montijo, Barreiro, PRT

**Keywords:** neurosyphilis, ocular neuritis, ocular syphilis, syphilis, treponema pallidum

## Abstract

Syphilis is transmitted via sexual contact and spreads through the lymphatic and hematologic systems. Ocular syphilis is an uncommon yet serious manifestation of syphilis that can cause significant vision impairment. This report presents a case of ocular syphilis in a 41-year-old otherwise healthy woman who presented with left-sided blurry vision and ocular discomfort. Examination confirmed decreased visual acuity and optic disc swelling in the left eye. Magnetic resonance imaging findings suggested optic neuritis. After ruling out other causes of optic neuritis and considering the presentation and diagnostic tests, including nontreponemal and treponemal, the diagnosis of ocular syphilis was confirmed. The patient was started on ceftriaxone and fully recovered.

## Introduction

Syphilis is a bacterial infection spread through sexual contact, caused by the spirochete *Treponema pallidum* [1]. In the early 2000s, due to disease prevention, early identification, and the use of penicillin, the incidence of syphilis plummeted. However, for the past two decades, there has been a global increase in the reported cases, particularly among men who have sex with men and people living with HIV, who are more susceptible to aggressive or atypical forms of the disease [1,2].

The infection usually comprises three stages (primary, secondary, and tertiary). The disease can present in various stages, and ocular syphilis can occur at any stage, though it is more commonly observed in tertiary syphilis [2,3]. After transmission and dissemination, a painless ulcer usually develops at the site of contact, which represents primary syphilis. The lesion heals after two to six weeks. Four to eight weeks following the ulcer, secondary syphilis with further dissemination occurs, including to the central nervous system [2]. Tertiary syphilis represents the final stage of infection and typically manifests years after the initial infection if left untreated [2,4].

Ocular syphilis is a rare but serious manifestation of syphilis, often associated with delayed diagnosis and potentially leading to irreversible vision loss. Recent studies also reported a surge in the incidence of ocular syphilis, which is related to the increasing prevalence of the infection. Eye involvement may result from direct invasion of the ocular structures by *T. pallidum *or immune-mediated inflammation following hematogenous dissemination [2,5].

Given the rising rates of syphilis and HIV co-infection, recognizing ocular presentations is critical. Clinicians must maintain a high level of suspicion for ocular syphilis, particularly in patients with visual symptoms and known risk factors, to ensure early diagnosis and treatment. This case is notable for illustrating optic nerve involvement in a non-HIV-infected individual, highlighting the need for clinical vigilance even in immunocompetent patients.

## Case presentation

A previously healthy 41-year-old woman from India presented with progressive left-sided blurred vision with visual acuity compromise. She also complained of discomfort in her left eye and photophobia. There was no history of trauma, and the patient denied other symptoms such as double vision, headache, dizziness, vertigo, speech disturbances, weakness, or loss of motor function.

Other symptoms such as diarrhea, arthralgia, jaundice, abdominal pain, fever, and weight loss were also denied. There was no recent history of viral or bacterial infection, as well as other illnesses.

She was not on regular medication and had no known allergies. She also denied alcohol, tobacco, or recreational drug use. There was no history of blood transfusion, high-risk sexual contact, or known exposure to sexually transmitted infections.

Previous medical history and family history were unremarkable for systemic or neurological disease. The patient was originally from India but had been living in Portugal for the previous five years and had not traveled elsewhere since.

Ophthalmic examination revealed a decreased visual acuity of 5/10 and mild conjunctival injection in the left eye. Fundoscopy after pharmacological mydriasis revealed optic disc swelling. Optical coherence tomography (OCT) findings were within normal limits; no significant structural abnormalities were detected in the retina, and there were no signs of optic nerve head swelling. There were no visible lesions around the eyes. There were no changes in the right eye.

Neurologic examination was remarkable for diminished visual acuity in the left eye, dyschromatopsia on the Ishihara test, and evaluation of visual fields revealed a left eye defect in the nasal field and a relative afferent pupillary defect in the left eye. Otherwise, there were no changes in speech or language, and eye movements were preserved with no diplopia or nystagmus. Facial sensibility was preserved, no facial asymmetries were present, and the facial muscle strength was preserved. No signs of meningism were noted.

The rest of the physical examination was innocent, with no lymphadenopathies, rashes, or signs of secondary syphilis.

**Figure 1:**
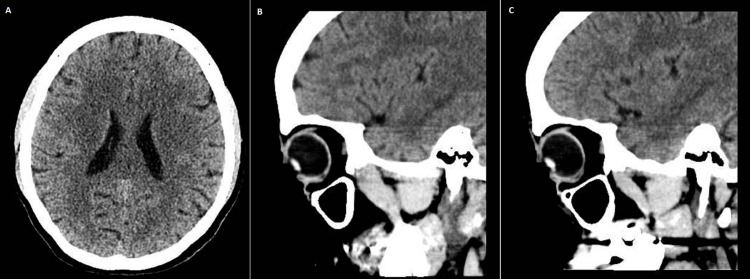
Brain (A), right orbit (B), and left orbit (C) CT scan with no abnormalities CT: computed tomography

A brain and orbit computed tomography (CT) scan was obtained, revealing a mild, unspecific reduction of the parietal encephalic volume bilaterally and no other changes (Figure 1).

Complete blood count, creatinine, urea nitrogen, electrolytes, liver, and thyroid function were normal. Folic acid and vitamin B12 levels were within normal range. There was no evidence of diabetes mellitus or dyslipidemia. Serologies for toxoplasmosis, cytomegalovirus, hepatitis, and HIV I/II were negative. Myelin oligodendrocyte glycoprotein (MOG) antibodies and aquaporin-4 antibodies were negative. Antineutrophil cytoplasmic antibodies and lupus anticoagulant were negative. Rapid plasma reagin (RPR) was positive with a titer of 32 dilutions. *T. pallidum* hemagglutination assay (TPHA) was positive. The patient denied any symptoms related to secondary syphilis. Cerebrospinal fluid (CSF) was clear with normal pressure, and the analysis revealed a slight increase in leukocytes (7/uL) and protein level (49 mg/dL). CSF glucose levels were normal, and the Venereal Disease Research Laboratory (VDRL) test was negative. CSF polymerase chain reaction for herpes simplex, cytomegalovirus, Epstein-Barr virus, human T-lymphotropic virus (HTLV)-1, and enterovirus was negative. Cultural exams were also negative.

**Figure 2:**
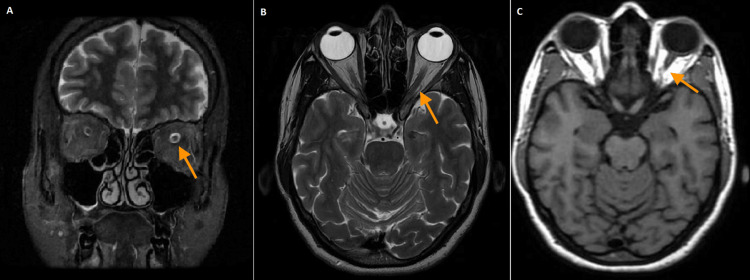
Brain and orbit MRI showing left optic nerve (orange arrows) hyperintensity in coronal STIR (A). Axial section T2 (B) and axial section T1 (C) demonstrate normal signal intensity of the left optic nerve MRI: magnetic resonance imaging; STIR: short tau inversion recovery sequence

A brain and orbit MRI was obtained as illustrated in Figure 2. Coronal short tau inversion recovery sequence and T2-weighted sequence revealed a focal signal alteration of the left optic nerve, characterized by relative hyperintensity in the intracanalicular segment, without appreciable thickening. The remaining segments of the optic nerve showed a normal signal, clearly distinguishable from the CSF. The signal, morphology, and volume of the optic chiasm were also normal. Vascular structures show regular flow. The remaining intraorbital structures were unremarkable. These findings were suggestive of left-sided optic neuritis.

The patient was started on ceftriaxone 2 g/day and completed a course of 14 days. She was reevaluated six weeks after treatment completion with a remarkable improvement in her left eye's visual acuity to 9/10. The pain in her left eye also resolved, and fundoscopy under pharmacologic mydriasis was normal in both eyes. The blood RPR showed a titer of 2 dilutions after treatment. The patient maintained her follow-up in the outpatient clinic.

## Discussion

We present the case of a female patient with left eye optic neuritis, resulting in impaired vision with decreased visual acuity and pain, who was diagnosed with ocular syphilis and had a notable recovery after treatment with ceftriaxone. MRI findings were suggestive of left-sided optic neuritis. The diagnosis was obtained through a positive RPR test with a positive TPHA test as confirmation and therapy responsiveness. Other causes of ocular neuritis were excluded.

Ocular syphilis is a form of neurosyphilis, and it is rare, especially as the primary manifestation. Its prevalence has increased in the context of the ongoing HIV epidemic. Early recognition is important, as untreated ocular syphilis can be potentially devastating and result in reduced vision and, in more severe cases, complete loss of vision [3,6]. We found this case to be of particular interest due to its presentation. Ocular syphilis is uncommon and rarely the first manifestation of the disease [2,3]. Our patient had no history of symptoms suggesting secondary syphilis. It is reported that many patients do not notice secondary manifestations [3].

Ocular syphilis can affect any structure of the eye, causing uveitis, retinitis, vasculitis, iritis, papillitis, keratic precipitates, vitritis, optic neuropathy, and perineuritis. Ocular syphilis can also affect the periocular tissues and the motor nerves [2,5]. In this case, optic disc swelling was noted, suggesting an inflammatory process in the posterior segment.

Optic neuritis related to syphilis lacks specific distinguishing features, and guidelines regarding the diagnosis of ocular syphilis are not unanimous. It has been described in all stages of infection, affecting multiple structures in one or both eyes. Therefore, the diagnosis is based on clinical presentation, serum, and CSF tests. Our patient's RPR titer of 1:32 dilutions falls within the range commonly observed in ocular syphilis, though titers can vary depending on disease stage and individual immune response [7,8]. Fundoscopy examination and OCT are useful tools for assessing retinal involvement. MRI is imperative to assess ocular nerve conditions [5,9].

Although clinical guidelines vary, classically, the treatment for all stages of syphilis includes penicillin [2,9,10]. However, recent studies point out that ceftriaxone is an alternative as plausible as penicillin, with the advantage of being administered in a single daily dose [10]. Also, the use of steroids is controversial and only recommended in some guidelines [2]. Our patient was treated with ceftriaxone so that she could continue treatment at home with our home hospitalization team. The once-a-day intravenous administrations are more manageable for the patient and healthcare professionals.

When ocular syphilis is detected early and treatment starts promptly, the prognosis is good, and most patients fully recover. However, in cases where either diagnosis or treatment is delayed, depending on the amount of damage caused to the ocular structures, there can be permanent deficits [1,9].

It remains uncertain if the neuritis was the first manifestation of syphilis in this patient, as it is a rare presentation. We must consider that the patient might have had manifestations of primary or secondary syphilis without realizing it. In fact, these symptoms resolve even without treatment; thus, many patients do not seek medical consultation [2,3,6].

## Conclusions

The diagnosis of ocular syphilis can be challenging and requires a high degree of clinical suspicion, especially when it is the first complaint. Global cases of syphilis continue to rise. Therefore, maintaining awareness of the disease and its diverse and sometimes rare clinical presentations is essential for early diagnosis and timely treatment. While our patient experienced excellent visual recovery, outcomes can vary significantly depending on the timing of intervention and the extent of optic nerve involvement. This variability underscores the need to maintain awareness of the disease for prompt recognition and management to minimize the risk of complications that can be devastating.
